# Family System and Trauma-Informed Care for Elder Abuse Consultation Team Research

**DOI:** 10.1093/geroni/igaf122.1427

**Published:** 2025-12-31

**Authors:** Elizabeth Bloemen, Sarah Tietz, Sarah Cox, Daniel Lindberg

**Affiliations:** University of Colorado, Aurora, Colorado, United States; University of Colorado School of Medicine, Aurora, Colorado, United States; University of Colorado Hospital, Aurora, Colorado, United States; University of Colorado School of Medicine, Aurora, Colorado, United States

## Abstract

Elder abuse consultation teams are a promising model of care with initial evidence showing both an improvement in the recognition of abuse and the care that is provided to patients. By using a family system and trauma-informed approach, our team has grounded both our direct care and our measurement in theory. Our team approaches elder abuse cases through the lens of a family system, with abuse impacting an entire family rather than a single victim-harmer dyad. To this end, we strive to identify other possible victims in the family system and put into place interventions that improve the resiliency of both the patient and the family. This impacts both our clinical care and measurement as some of our critical outcomes include the identification of other potential victims and resources implemented at discharge. We also prioritize trauma-informed care for all members of the family system and reflect this in our research priorities through tracking of patient-centered discharge planning and harm reduction utilization. Through this session, we will describe the patient, family, and system-level variables we have measured throughout our 5 years of elder abuse consultations and the theoretical framework these are based upon. Initial data will be reported within this framework.

